# Soluble Urokinase-Type Plasminogen Activator Receptor and Inflammatory Biomarker Response with Prognostic Significance after Acute Neuronal Injury – a Prospective Cohort Study

**DOI:** 10.1007/s10753-024-02185-1

**Published:** 2024-11-14

**Authors:** Antti Sajanti, Santtu Hellström, Carolyn Bennett, Abhinav Srinath, Aditya Jhaveri, Ying Cao, Riikka Takala, Janek Frantzén, Fredrika Koskimäki, Johannes Falter, Seán B. Lyne, Tomi Rantamäki, Jussi P. Posti, Susanna Roine, Miro Jänkälä, Jukka Puolitaival, Sulo Kolehmainen, Romuald Girard, Melissa Rahi, Jaakko Rinne, Eero Castrén, Janne Koskimäki

**Affiliations:** 1 Neurocenter, Department of Neurosurgery, Turku University Hospital and University of Turku, P.O. Box 52, Hämeentie 11, FI-20521 Turku, Finland; 2 Neurovascular Surgery Program, Section of Neurosurgery, The University of Chicago Medicine and Biological Sciences, 5841 S. Maryland, Chicago, IL 60637 USA; 3 Department of Radiation Oncology, Kansas University Medical Center, Kansas City, KS 66160 USA; 4 Perioperative Services, Intensive Care and Pain Medicine, Turku University Hospital and University of Turku, POB 52, 20521 Turku, Finland; 5 Neurocenter, Acute Stroke Unit, Turku University Hospital, P.O. Box 52, FI-20521 Turku, Finland; 6 Department of Neurosurgery, University Medical Center of Regensburg, Regensburg, Germany; 7 Department of Neurosurgery, Brigham and Women’s Hospital, Harvard Medical School, Boston, MA USA; 8 Laboratory of Neurotherapeutics, Molecular and Integrative Biosciences Research Programme, Faculty of Biological and Environmental Sciences and Drug Research Program, Division of Pharmacology and Pharmacotherapy, Faculty of Pharmacy, University of Helsinki, Helsinki, Finland; 9 Department of Neurosurgery, Oulu University Hospital, Box 25, 90029 OYS Oulu, Finland; 10 Neuroscience Center, HiLIFE, University of Helsinki, Box 63, 00014 Helsinki, Finland

**Keywords:** Brain injury, Inflammation, SuPAR, Stroke, TBI, Outcome, Hemorrhage

## Abstract

Aneurysmal subarachnoid hemorrhage (aSAH), ischemic stroke (IS), and traumatic brain injury (TBI) are severe conditions impacting individuals and society. Identifying reliable prognostic biomarkers for predicting survival or recovery remains a challenge. Soluble urokinase type plasminogen activator receptor (suPAR) has gained attention as a potential prognostic biomarker in acute sepsis. This study evaluates suPAR and related neuroinflammatory biomarkers in serum for brain injury prognosis. This prospective study included 31 aSAH, 30 IS, 13 TBI, and three healthy controls (*n* = 77). Serum samples were collected on average 5.9 days post-injury, analyzing suPAR, IL-1β, cyclophilin A, and TNFα levels using ELISA. Outcomes were assessed 90 days post-injury with the modified Rankin Scale (mRS), categorized as favorable (mRS 0-2) or unfavorable (mRS 3-6). Statistical analyses included 2-tailed t-tests, Pearson’s correlations, and machine learning linear discriminant analysis (LDA) for biomarker combinations. Elevated suPAR levels were found in brain injury patients compared to controls (*p* = 0.017). Increased suPAR correlated with unfavorable outcomes (*p* = 0.0018) and showed prognostic value (AUC = 0.66, p = 0.03). IL-1β levels were higher in the unfavorable group (*p* = 0.0015). LDA combinatory analysis resulted a fair prognostic accuracy with canonical equation = 0.775[suPAR] + 0.667[IL1-β] (AUC = 0.77, OR 0.296, sensitivity 93.1%, specificity 53.1%, *p* = 0.0007). No correlation was found between suPAR and CRP or infection status. Elevated suPAR levels in acute brain injury patients were associated with poorer outcomes, highlighting suPAR’s potential as a prognostic biomarker across different brain injury types. Combining IL-1β with suPAR improved prognostic accuracy, supporting a multimodal biomarker approach for predicting outcomes.

**Supplementary Information:**

The online version contains supplementary material available at 10.1007/s10753-024-02185-1.

## Introduction

Aneurysmal subarachnoid hemorrhage (aSAH), traumatic brain injury (TBI), and ischemic stroke (IS) are prevalent and devastating conditions, imposing significant burdens on patients, their families and society [1, 2]. Inflammation plays a critical role in the progression of these neurological disorders, contributing to poorer outcomes and hindering neurological recovery [3–5]. The neuroimmunological response to brain injuries is complex, involving both pro-inflammatory and anti-inflammatory mediators that can either exacerbate damage or promote repair and regeneration [6–9].

Following acute brain injuries, mitochondrial dysfunction and neuroinflammation drive diffuse damage and chronic neurodegeneration [6–9]. In both ischemic and hemorrhagic strokes, impaired cerebral blood flow or vascular rupture results in metabolic insufficiency, exacerbating brain damage [8, 9]. Microglia and brain-infiltrating macrophages produce neuroinflammatory cytokines and reactive oxygen species (ROS), disrupting homeostatic functions such as immunosurveillance and phagocytosis [6–9]. This inflammatory response, involving various cells and pro-inflammatory mediators, not only exacerbates acute brain damage but also impedes recovery [4, 10]. Further complicating the prognosis after aSAH is the well-acknowledged complication of vasospasm, which has been notably linked to inflammatory processes [11].

Urokinase-type plasminogen activator receptor (uPAR) is a glycosylphosphatidylinositol-linked membrane protein predominantly found in immunologically active cells, such as T-lymphocytes and macrophages, but also in endothelial and smooth muscle cells [12]. Upon cleavage, the membrane-bound uPAR yields its soluble (s) form, suPAR. Previously, suPAR has been leveraged as a prognostic marker for septic infections, a variety of inflammatory diseases and pancreatic diseases [13–15]. Elevated urinary suPAR levels have been correlated with poor prognosis in patients with pancreatic ductal carcinoma [16] and preoperatively elevated serum suPAR levels have been linked to differentiating between malignant and non-malignant pancreatic lesions [15]. Elevated serum suPAR, together with c-reactive protein (CRP), has also been shown to correlate with the severity of acute pancreatitis [17]. Furthermore, elevated serum suPAR levels have been associated with increased stage of diabetic nephropathy [18]. Serum suPAR, together with other inflammatory markers IL-6, IL-16, CRP, and CCL3, was associated to the severity of sepsis-induced acute kidney injury [19]. Serum suPAR, along with other inflammatory biomarkers such as CRP, IL-6, and TNFα, correlated with the severity of chronic systemic inflammation [20]. High serum suPAR concentration predicted worse outcomes after COVID-19 [21]. Moreover, elevated suPAR concentrations in serum samples have shown associations with a spectrum of vascular pathologies [22, 23]. One previous study demonstrated that serum suPAR levels were correlated with aneurysmal subarachnoid hemorrhage (aSAH) compared to healthy controls, and elevated serum suPAR levels were associated with an increased risk of delayed cerebral ischemia [24]. These previous findings about suPAR underline its crucial role in the mechanism of various inflammatory processes.

To explore the prognostic value of inflammatory biomarkers in acute brain injuries, we selected suPAR, tumor necrosis factor alpha (TNFα), interleukin-1β (IL-1β), and cyclophilin A for our study. SuPAR was chosen as our primary candidate due to its established role in various inflammatory and vascular pathologies, and we hypothesized that suPAR could serve as both a prognostic and diagnostic marker in acute brain injuries. TNFα and IL-1β were included because they are widely studied inflammatory molecules, well-known for their significant roles in mediating neuroinflammation and neuronal damage [25–33]. Cyclophilin A was selected due to its critical involvement in inflammatory pathways and its inhibition by cyclosporine, which has been studied in TBI contexts for its therapeutic potential in reducing cyclophilin A levels [34]. These biomarkers were also validated through Reactome network analysis [35], which addressed their interconnected biological roles and synergistic effects.

## Materials and Methods

### Study Design and Participants

**Fig. 1:**
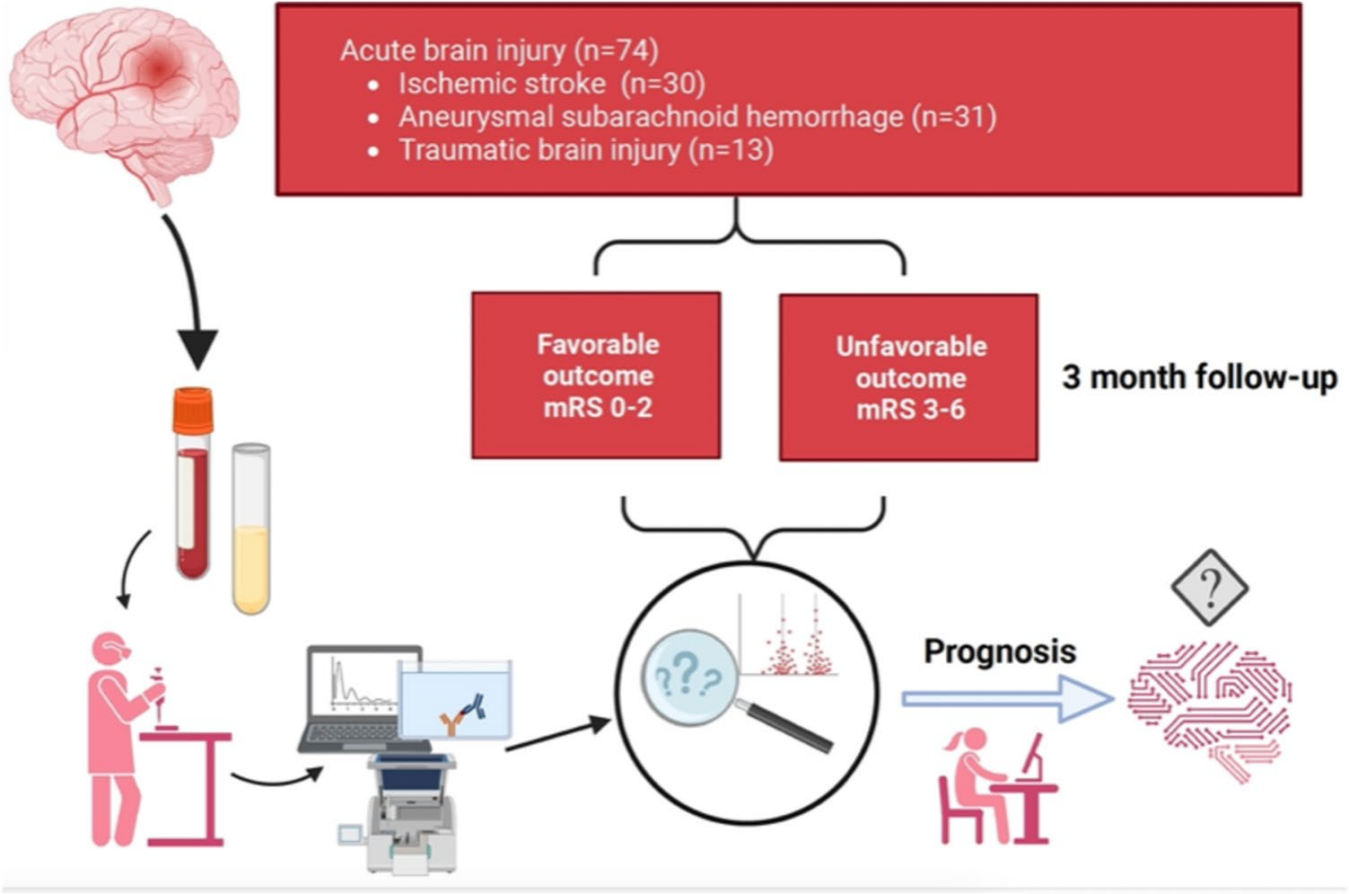
Flow chart of the study. The prospective cohort (*n* = 74) consisted consecutively collected patients of ischemic stroke (IS) (*n* = 30), aneurysmal subarachnoid hemorrhage (aSAH) (*n* = 31), traumatic brain injury (TBI) (*n* = 13) patients and healthy controls (*n* = 3). Blood samples were drawn, and serum was used in analyses. ELISA method was used to detect biomarker levels (suPAR, TNFα, IL-1β, cyclophilin a). Patients were categorized based on their modified Rankin scale (mRS) scores into favorable (mRS = 0-2) and unfavorable (mRS = 3-6) outcome groups.

This prospective cohort study enrolled 74 consecutively admitted patients, comprising individuals with IS (*n* = 30), aSAH, (*n* = 31), and TBI, (*n* = 13) (Fig. 1). Inclusion criteria were aneurysmal subarachnoid hemorrhage, ischemic stroke (embolic, thrombotic, or cryptogenic), or traumatic brain injury resulting in subdural hematoma requiring surgical evacuation. Eligible participants were over 18 years of age and provided informed consent. These patients were admitted to the tertiary care University Hospital of Turku, Finland, between 2016 and 2019 and received standard clinical treatment following in-house protocol aligned with treatment recommendations for aSAH, IS and TBI management [36–38]. Infection was defined as diagnosis (ICD-10) of bacterial infection, with initiated antibiotic treatment for the infection. If antibiotics were administered prophylactically that was not counted as infection. Eleven patients declined to give consent for the study. One patient initially gave consent but later withdrew resulting in their samples and other data being excluded from the study. Lastly, six enrolled patients were excluded from the protein biomarker detection measurements due to the availability of only early samples (1-2 days post-insult). No study patients were lost to follow-up. In addition, three (*n* = 3) healthy control patients were included with no neurological diseases.

### Serum Sample Preparation

Blood samples were taken at 5.9 ± 2.2 (SD) days after the insults. Standard 10 ml venous blood serum collection tubes (BD Vacutainer No Additive, REF 364915) were utilized for blood collection. Following the blood draw, each sample was allowed to rest at room temperature for 30 to 60 minutes to allow for the clot to form. The serum was then isolated by centrifuging the blood sample at the end of the clotting time in a horizontal rotor (swing-out head) for 15 minutes at 2200 g at room temperature. Subsequently, the serum was aliquoted in three 10 ml clean serum collection tubes for storage at −80 °C.

### Sample Analytics

To explore the prognostic value of inflammatory biomarkers in acute brain injuries, we selected soluble urokinase-type plasminogen activator receptor (suPAR), tumor necrosis factor alpha (TNFα), interleukin-1β (IL-1β), and cyclophilin A for our study as described in the introduction. SuPAR was chosen as our primary candidate due to its established role in various inflammatory and vascular pathologies. We hypothesized that suPAR could serve as both a prognostic and diagnostic marker in acute brain injuries. Biomarkers were analyzed with curated Reactome FIviz network analysis program [35], which highlighted their potential synergistic roles in functional network (input proteins uPAR. IL-1β, Cyclophilin A and TNFα) (Supplemental fig. S1, supplemental material). SuPAR, TNFα, IL-1β, and cyclophilin A concentrations were measured in serum samples using the commercially available enzyme-linked immunosorbent assay (ELISA) (Invitrogen®, Catalog numbers: EHPLAUR, EH138RB, BMS224-2, KHC3011). Assay range and analytical sensitivity were for each molecule: suPAR (16.38-4000 pg/ml; 15 pg/ml), Cyclophilin A (2.05-500 ng/ml; 2,05 ng/ml), IL-1β (3.9-250 pg/ml; 0.3 pg/ml), TNFα (15.6-1000 pg/ml; 1.7 pg/ml). The protocol was performed as per manufacturer’s guidelines. The samples were thawed only once. An experienced researcher performed loading of the wells and was blinded to the patients’ outcomes. All the samples were loaded in duplicates and averaged. Measurements were performed with a Varioskan® Flash analyzer running SkanIt Software version 2.4.3 RE. Four-parameter logistic regression analysis was performed to estimate the sample concentration.

### Evaluation of Functional Outcome

The outcomes for aSAH patients were assessed during a three-month structured follow-up at the outpatient clinic. For IS and TBI patients, outcomes were evaluated *via* structured telephone interviews. The modified Rankin Scale (mRS) was used to determine outcomes, dividing the cohort into favorable (mRS 0-2) and unfavorable (mRS 3-6) subgroups.

### Statistical Methods

We conducted a two-tailed t-test to compare serum biomarker levels (suPAR, IL-1β, Cyclophilin A, and TNFα) across patient outcomes. Differences in measured protein biomarker concentrations between disease groups were analyzed with one-way ANOVA. Pearson’s correlation coefficients were calculated to explore the linear relationships between these biomarkers. The Chi-squared test or Fisher’s exact test was used to calculate *p* values for categorical variables. Additionally, multivariate linear canonical discriminant analysis (LDA) was performed, and canonical scores were used to build a combinatory biomarker with logistic modeling predicting the outcome [39, 40]. The Youden method was applied to determine the best sensitivity and specificity in receiver operating characteristic (ROC) analytics [41]. Areas under the curve (AUCs) ranging from 0.6 to 0.7, 0.7 to 0.8, 0.8 to 0.9, and greater than 0.9 were classified as acceptable, fair, good, and excellent for discrimination, respectively [42]. A significance level of *p* < 0.05 was set for statistical tests. When candidate biomarkers were selected into a combinatory model, only molecules passing Bonferroni correction *p* < 0.05 was selected into a final model [43]. We identified and excluded outliers using the ROUT method, with a false discovery rate set at *Q* = 1% [44]. All data analyses were conducted using SAS 9.4 (SAS Institute Inc., 2016, Cary, NC, US) and Prism 9.4.1 (GraphPad Software, LLC).

## Results

### Patient Demographics and Clinical Characteristics

The cohort consisted of 31 (41.9%), 13 (17.6%), and 30 (40.5%) patients respectively, totaling 74 patients. Demographic analysis revealed a slightly higher proportion of male patients (39/74, 52.7%) (Supplemental table S1). The age distribution in the cohort was between 23 and 75 years while the mean age of the patients was 58.4 ± 12.7 years. Of the patients, 54.1% (40/74) of the cohort had a favorable outcome (mRS 0-2) while 45.9% (34/74) had an unfavorable outcome (mRS 3-6). Among patients with a favorable outcome the division between different brain injuries was 17 aSAH (54.8%), 19 IS (63.3%) and 4 TBI (30.8%) patients. The division between patients with unfavorable outcomes were 14 aSAH (45.2%), 11 IS (36.7%) and 9 TBI (69.2%) (Supplemental table S1). Favorable and unfavorable groups compared were matched sex (*p* = 0.98). Patients in the unfavorable outcome group were older (*p* = 0.03) (Table 1). Further analysis of age and suPAR concentrations confirmed that suPAR did not correlate with age (*r* = 0.03010, *R*^2^ = 0.0009, *p* = 0.7990). Additionally, age did not correlate with suPAR in either outcome group: favorable (*r* = 0.03790, *R*^2^ = 0.0014, *p* = 0.8164) or unfavorable (*r* = −0.0448, *R*^2^ = 0.0020, *p* = 0.8013) (Table 1, Supplemental fig. S2). IL-1β showed a trend toward negative correlation (*r* = −0.01657, *R*^2^ = 0.02745, *p* = 0.1673) (Table 1, Supplemental fig. S2).

**Table 1:** Patient characteristics and concentrations of soluble urokinase-type plasminogen activator receptor (suPAR), interleukin-1β, cyclophilin a and tumor necrosis factor α (TNFα) from the acute brain injury cohort (*n* = 74) and healthy individuals (*n* = 3). Modified Rankin scale (mRS): Favorable 0-2, unfavorable mRS 3-6. Two-sample t-test (continuous) or chi square test or Fisher’s exact test (categorical) for *p* values

Variables	Favorable (*n* = 40)	Unfavorable (*n* = 34)	*p*-value
Age* in years			0.031
Mean±SD	55.5±12.0	61.9±12.8	
Min–Max	23.0–74.0	30.0–75.0	
Median (IQR)	56.5 (46.3–65.0)	66.5 (52.0–71.3)	
Sex			0.98
Male	21 (52.5)	18 (52.9)	
Female	19 (47.5)	16 (47.1)	
Type of brain injury			0.14
aSAH	17 (54.8)	14 (45.2)	
TBI	4 (30.8)	9 (69.2)	
IS	19 (63.3)	11 (36.7)	
suPAR (ng/ml)			0.0018
Mean±SD	2.84±1.06	4.56±2.81	
Min–Max	0.94–5.47	1.09–11.42	
Median (IQR)	2.75 (2.06–3.58)	3.83 (2.26–6.96)	
IL-1β (pg/ml)			0.0015
Mean±SD	15.70±10.55	26.70±16.72	
Min–Max	4.18–39.35	3.93–73.28	
Median (IQR)	14.64 (6.60–20.44)	27.10 (10.32–36.74)	
Cyclophilin A (ng/ml)			0.14
Mean±SD	64.57±117.60	110.50±120.40	
Min–Max	0–427.60	0–349.90	
Median (IQR)	2.88 (0–53.62)	52.77 (0.21–236.50)	
TNFα (pg/ml)			0.39
Mean±SD	20.23±4.39	19.29±4.87	
Min–Max	10.04–28.10	10.27–27.44	
Median (IQR)	20.12 (17.50–23.47)	19.23 (15.36–22.39)	
suPAR (ng/ml)	Healthy (*n* = 3)		0.018†
Mean±SD	0.47±0.12		
Min–Max	0.37–0.60		
Median (IQR)	0.45 (0.37–0.60)		
Age in years			0.04‡
Mean±SD	47.33±19.09		
Min–Max	33.0-69.0		
Median (IQR)	40.00 (33.00–69.00)		
Sex			0.94‡
Male	1 (33.33)		
Female	2 (67.67)		

* suPAR and IL-1β levels did not correlate with age

† Statistical comparisons of suPAR between healthy *versus* favorable plus unfavorable outcome groups

‡Statistical comparisons of age and sex between healthy *versus* favorable plus unfavorable outcome groups

### suPAR Concentration Associate with Outcome after Brain Injury

**Fig. 2:**
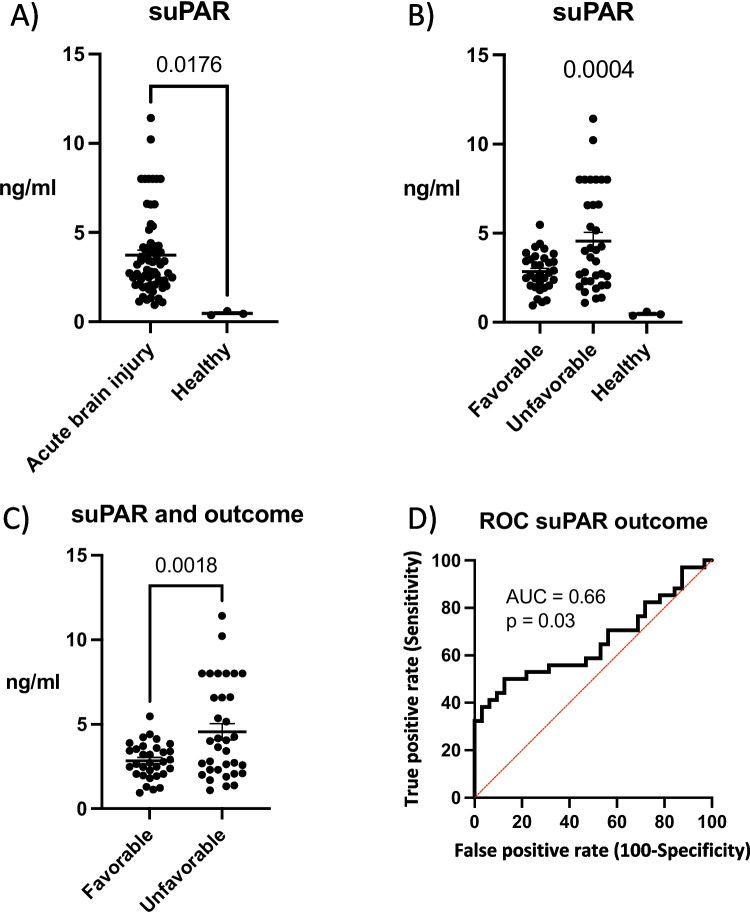
Soluble urokinase-type plasminogen activator receptor (suPAR) concentrations in acute after brain injury and healthy controls. **A**) Significantly higher suPAR concentrations were detected in acute brain injury group *versus* healthy controls (*p* = 0.0176) **B**) favorable outcome, unfavorable outcome and healthy control group were significantly different in suPAR concentration (*p* = 0.0004) (ANOVA) **C**) higher suPAR concentration was associated with unfavorable outcome (*p* = 0.0018). **D**) Receiver operating characteristic analysis (ROC) showed acceptable prognostic accuracy (area under the curve = 0.66, *p* = 0.03, 95% CI 0.52-0.79). Favorable outcome = 0-2 modified Rankin scale (mRS), favorable outcome = 3-6 mRS. acute brain injury = ischemic stroke, aneurysmal subarachnoid hemorrhage and traumatic brain injury groups combined. Two-tailed 2-sample t-test. Panel B ANOVA. Data represent mean ± SEM.

We observed a significant increase in suPAR levels among patients with acute brain injuries compared to healthy controls (*p* = 0.0176) (Fig. 2A and Table 1). Patients with unfavorable outcome (mRS scores of 3-6) had higher suPAR concentrations (*p* = 0.0018) (Fig. 2B and C). Receiver operating characteristics (ROC) analysis demonstrated that suPAR has an acceptable prognostic value, with an area under the curve (AUC) of 0.66 (*p* = 0.03, 95% CI 0.52-0.79) (Fig. 2D). When we studied suPAR levels between diseases, we did not find any statistically significant differences between the disease groups (IS, aSAH, and TBI) (favorable outcome group *p* = 0.30, unfavorable outcome group (*p* = 0.33) (Supplemental Fig. 3, supplemental material).

### Infection Status and suPAR

**Fig. 3:**
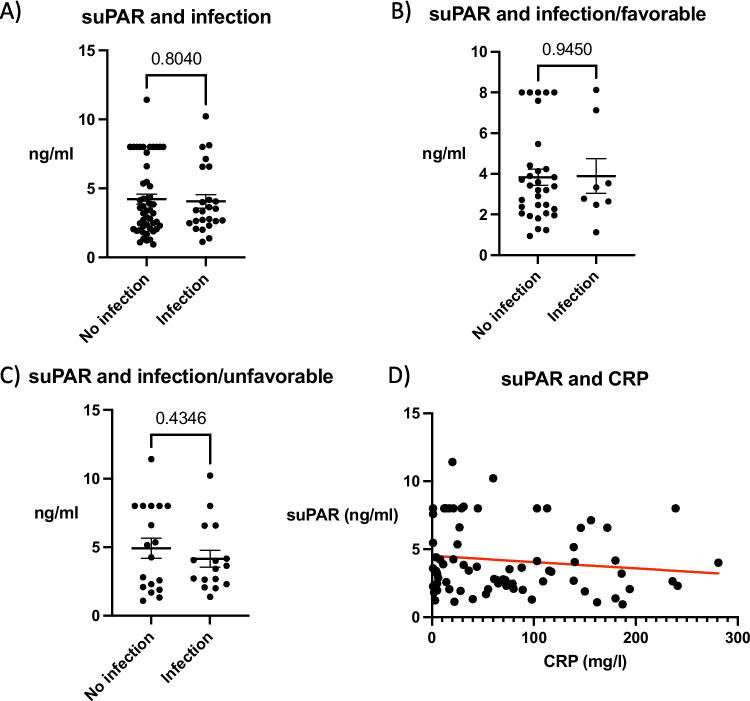
Analysis of soluble urokinase-type plasminogen activator receptor (suPAR) levels and infection status in brain injury outcomes. **A**) no significant association between suPAR levels and infection status observed across the entire cohort (*p* = 0.8040). **B**) within the favorable outcome group, suPAR levels show no association with infection status (*p* = 0.9450). **C**) Examination of the unfavorable outcome group also indicates a lack of association between suPAR levels and infection status (*p* = 0.4346). **D**) SuPAR did not correlate with C-reactive protein levels (*p* = 0.2680, *r* = −0.1304, *R*^2^ = 0.0170). Favorable outcome = 0-2 modified Rankin scale (mRS), favorable outcome = 3-6 mRS. two-tailed 2-sample t-test, data represent mean ± SEM. Pearson correlation analysis in panel **D.**

In our investigation into the relationship between suPAR levels and infection status, we found no significant association (Fig. 3). Analysis showed that suPAR levels were similar irrespective of the infection status (*p* = 0.8040) (Fig. 3A). This finding was consistent when the data were segregated into outcome groups. In the favorable outcome group, suPAR levels showed no significant association with infection status (*p* = 0.9450) (Fig. 3B). Similarly, within the unfavorable outcome group, there was no significant association between suPAR levels and infection status (*p* = 0.4346) (Fig. 3C). Further, our results indicated no correlation between suPAR concentrations and the highest C-reactive protein (CRP) levels (*p* = 0.2680, *r* = −0.1304, *R*^2^ = 0.0170) (Fig. 3D).

### Circulating Inflammatory Biomarker Response after Brain Injury

**Fig. 4:**
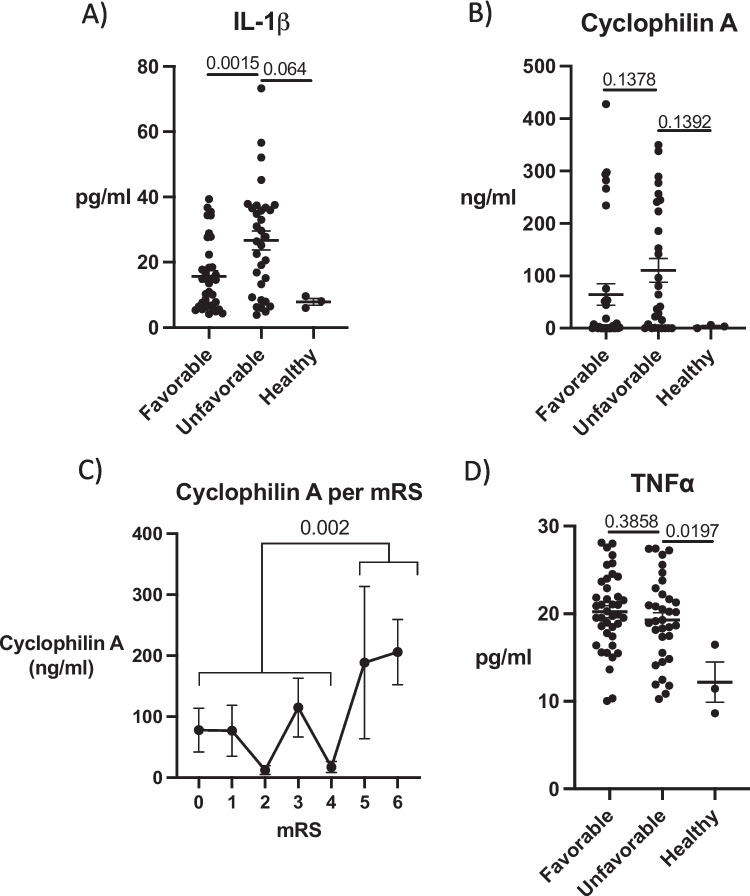
Brain injury induced neuroinflammatory biomarkers in brain injury outcomes. **A**) IL-1β was increased in unfavorable patient group compared to favorable outcome and healthy (*p* = 0.0015 and 0.064, respectively). **B**) Cyclophilin a showed a notable upward trend in patients with unfavorable outcomes (*p* = 0.1378), and similarly compared to healthy (*p* = 0.1392). **C**) Cyclophilin a was significantly higher in the mRS groups five and six (*p* = 0.002). **D**) TNFα levels remained consistent across different outcome groups (*p* = 0.3858), but in healthy group TNFα concentrations were significantly lower (*p* = 0.0197). Two-tailed 2-sample t-test, data represent mean ± SEM.

After evaluating the associations between suPAR, infection status, and CRP, we expanded our analysis to encompass a broader range of inflammatory biomarkers and their potential change in response following brain injury. IL-1β was increased in unfavorable patient group compared to favorable outcome and healthy (*p* = 0.0015 and 0.064, respectively) (Fig. 4A, Table 1). Regarding Cyclophilin A, the data showed an upward trend in concentrations among patients with unfavorable outcomes (*p* = 0.1378), indicating a potential association with more severe injury cases (Fig. 4C). Furthermore, when compared to healthy individuals, Cyclophilin A demonstrated an increasing trend, but not statistically significant (*p* = 0.1392). In contrast, TNFα levels did not exhibit substantial variation between different outcome groups (*p* = 0.3858), suggesting a more consistent expression irrespective of injury severity (Fig. 4D). However, when comparing TNFα concentrations in the healthy group, we noted significantly lower levels (*p* = 0.0197), indicating a potential baseline difference in the context of brain injuries.

### Combinatory Prognostic Model

Our investigation into the potential of a combinatory prognostic model for brain injury outcomes focused on integrating statistically significant IL-1β and suPAR (*p* < 0.05, Bonferroni corrected) biomarkers. Despite IL-1β’s significant association with unfavorable outcomes and its fair performance in the AUC analyses, it is noteworthy that IL-1β and suPAR were not co-correlated (*r* = 0.09, *p* = 0.48, *R*^2^ = 0.008). This lack of correlation suggests that each biomarker may contribute unique and independent information to the prognosis of brain injury outcomes.

**Fig. 5:**
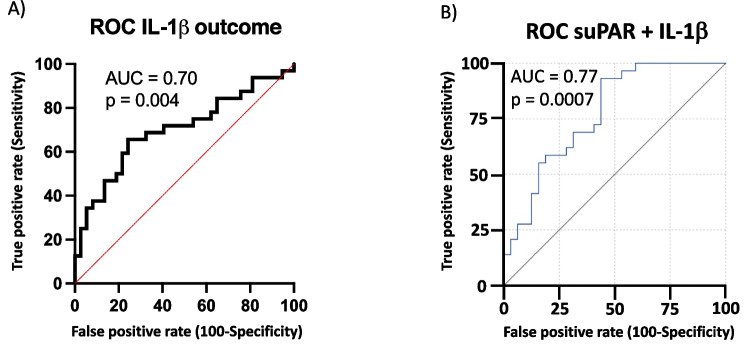
Prognostic performance of the IL-1β and combinatory suPAR biomarker. **A**) Receiver operating characteristic (ROC) analysis showed satisfactory prognostic accuracy (area under the curve (AUC) = 0.70, *p* = 0.004, 95% CI 0.57-0.83). **B**) Linear discriminant analysis (LDA) of soluble urokinase-type plasminogen activator receptor (suPAR) and interleukin-1β (IL-1β) showed a good and improved model prognosing favorable outcome (OR 0.296, 95% CI 0.147-0.597; AUC = 0.77, 95% CI 0.66-0.89; sensitivity 93.1%, specificity = 53.1%, *p* = 0.0007). LDA of combinatory biomarker resulted an equation with canonical scores: 0.775[suPAR] + 0.667[IL1β].

In the independent ROC analysis, IL-1β showed a fair level of prognostic accuracy with an AUC of 0.70 (*p* = 0.004, 95% CI = 0.57-0.83) (Fig. 5A). The machine learning algorithm linear discriminant analysis combining these biomarkers demonstrated improved predictive power for favorable outcome: OR 0.296 (95% CI = 0.147-0.597); AUC 0.77 (95% CI = 0.66-0.89), *p* = 0.0007, with 93.1% sensitivity and 53.1% specificity (Youden J = 0.462) (Fig. 5B). Furthermore, the LDA formula, utilizing canonical scores of suPAR and IL-1β, provides a quantified assessment of their combined influence on prognostic predictions:

$$\textrm{Canonical}\ \textrm{score}=0.775\left[\textrm{suPAR}\right]+0.667\left[\textrm{IL}-1\upbeta \right].$$

These findings affirm the potential of using a multimodal biomarker approach in clinical settings to enhance the precision of outcome predictions in patients even with different types of brain injuries.

## Discussion

This study aimed to elucidate the prognostic value of serum suPAR levels and associated neuroinflammatory biomarkers in patients with acute brain injuries, including aSAH, IS, and TBI. Elevated suPAR levels were consistently associated with unfavorable outcomes, supporting the notion that suPAR serves as a global marker of inflammation and immune activation, potentially reflecting the severity and prognosis of brain injuries. The association between increased serum suPAR levels and unfavorable outcomes across different types of brain injuries underscores the potential of suPAR as a promising biomarker for assessing prognosis in acute brain injuries. This is particularly noteworthy as the search for reliable biomarkers that can predict outcomes across various types of brain injuries has been challenging. The moderate prognostic value of suPAR suggests that suPAR could be an important component of a multimodal biomarker strategy to refine outcome predictions in acute brain injury.

We also observed elevated suPAR levels in all disease groups compared to healthy controls. Previous studies have reported suPAR levels in healthy populations ranging from 0.5 to 4 ng/ml, depending on the measurement method, which aligns with our study’s healthy control group mean of 0.47 ng/ml (Table 1). Although the control group size was limited, these preliminary findings suggest a potential role for suPAR as a distinguishing marker in brain injury. Previous studies have shown that suPAR levels may increase with age [45–47]. Notably, age was not associated with higher suPAR levels in our outcome analysis (Fig. S2), supporting the utility of suPAR as an independent biomarker in this context. Nonetheless, future studies with larger control groups will be necessary to accept suPAR’s diagnostic potential.

### suPAR Associating with Outcome

SuPAR is a promising prognostic biomarker in acute brain injuries such as TBI and IS [22, 48, 49]. Elevated serum and cerebrospinal fluid suPAR levels have also been observed in patients with aSAH compared to healthy controls [24]. However, there are not many studies on suPAR and related brain injuries; suPAR has been studied more widely in the context of general inflammatory processes and cancer [14–16, 19, 20, 45] (Table 2).

**Table 2:** Soluble urokinase-type plasminogen activator receptor (suPAR) studies and associations with outcome and inflammatory biomarkers

Disease studied	Association with outcome	Association with inflammatory molecules	Reference
Acute kidney injury and sepsis	Yes	IL-6, IL-16, CRP, CCL3	Nusshag et al. [19]
Diabetic nephropathy	Yes	–	Wu et al. [18]
Systemic chronic inflammation	Yes	CRP, IL-1b, IL-6, IL-8, IL-10, IL-18, MCP-1, CCL2, TNFα	Rasmussen et al. [20]
Cardiovascular diseases	Yes	CRP, IL-6	Hodges et al. [23]
SAH	No	CRP	Kiiski et al. [50]
Pancreatitis	Yes	CRP	Zhang et al. [17]
Pancreatic ductal adenocarcinoma	Yes	–	Sorio et al. [16]
COVID-19	Yes	PTX3, CD14	Zhan et al. [21]
Ischemic stroke and TIA	Yes	CRP, Procalcitonin, IL-6, IL-17, IL-10	Śmiłowska et al. [49]; Onatsu et al. [22]
Traumatic brain injury	Yes	–	Yu et al. [48]
Chronic kidney disease	Yes	high-sensitivity CRP, CD40	Hayek et al. [51]

Our study found no correlation between serum suPAR and CRP levels, which is consistent with an earlier studies reporting no correlation between plasma suPAR levels and CRP, IL-6, or IL-10 in patients with severe sepsis [52]. There is evidence indicating that high-sensitivity CRP is associated with higher suPAR levels in chronic kidney disease, where high-sensitivity CRP reflects low-grade, chronic inflammation, while normal CRP typically signals more acute inflammatory responses. [51]. Interestingly, weak correlation between suPAR and myeloperoxidase suggests that suPAR may reflect general immune system activation rather than directly mediating inflammatory actions [52]. SuPAR has also been associated with other inflammatory processes such as systemic chronic inflammation, various cardiovascular diseases, pancreatitis, pancreatic carcinoma and diabetic nephropathy [16–18, 20, 23]. These associations underscore the significant role of suPAR in inflammation processes. Notably, there are other studies reporting an association with CRP (Table 2), indicating that the relationship between CRP and suPAR is multifactorial and likely not direct association.

Contrastingly, the findings by Kiiski et al. revealed no significant association between plasma suPAR levels and neurological outcomes in aSAH patients [50]. Several factors might contribute to these divergent findings. Firstly, the difference in patient populations and injury types between the two studies could account for the variability in suPAR’s prognostic utility. While our study spanned a broad spectrum of acute brain injuries, Kiiski et al. [50] focused exclusively on aSAH patients. However, we did not find any differences in suPAR concentration between aSAH, TBI, and IS. Moreover, the methodology of suPAR measurement could influence its prognostic value. Our study assessed serum suPAR levels, whereas Kiiski et al. [50] measured plasma concentrations. In our cohort, the mean suPAR level in the favorable outcome groups was 2.84 ng/ml, which is in line with their measurements. However, in the unfavorable outcome group, our findings differed from those of Kiiski et al., with our cohort having mean concentration of 4.56 ng/ml compared to their 2.66 ng/ml.

In our study, machine learning LDA model incorporating suPAR and IL-1β demonstrated a promising prognostic performance for predicting favorable outcomes, with an OR of 0.296. Notably, the model exhibited very high sensitivity (93.1%) but moderate specificity (53.1%). This is particularly advantageous feature for a prognostic biomarker as the high sensitivity means that unfavorable patients are not loosely identified, reducing the risk of misclassifying favorable outcome patients as unfavorable. This ensures that possible clinical decisions, such as withdrawing treatment based on poor prognosis, are more accurate. The canonical equation derived from the LDA, 0.775[suPAR] + 0.667[IL1β], underscores the combined biomarker’s potential utility in clinical settings where accurate prognostication is essential for optimizing patient outcomes.

### Circulating Inflammatory Molecules after Neuronal Injury

The interaction between Cyclophilin A and apoptosis-inducing factor (AIF) has been shown to promote neuronal injury in neonatal mice models after ischemic brain injury [53]. Inhibition of this cascade reduced apoptotic processes following ischemic injury [54]. In aSAH models Cyclophilin A is increased activating the CD147 receptor and NF-κB inflammatory signaling pathway, which can also induce blood-brain barrier disruption [55]. Our findings regarding Cyclophilin A align with these previous studies, as Cyclophilin A levels showed an upward trend in the unfavorable patient group, though the change was not statistically significant (*p* = 0.1392).

There is marked evidence in the literature supporting that IL-1β is a crucial inflammatory mediator in neuronal injury of stroke and TBI [30]. IL-1β is a key inflammatory mediator after TBI in mice models. Neutralization of IL-1β improved cognitive outcomes in TBI mice models [55]. In our study, IL-1β was significantly increased in the unfavorable patient group, highlighting its important role in neuroinflammatory processes and its prognostic value after acute brain injury, and interestingly irrespective of the type of brain injury. The lack of a correlation between IL-1β and suPAR suggests that these biomarkers may reflect different aspects of the neuroinflammatory response, potentially offering a broader understanding of the underlying pathophysiological processes.

TNFα was found to be upregulated and induce neuronal apoptosis *via* p53 activation after TBI in mice [56]. Higher TNFα and IL-6 levels were also measured in the cerebrospinal fluid of patients with aSAH, compared to healthy controls [57]. In our study, there was no statistically significant difference in serum TNFα levels between the unfavorable and favorable outcome groups. This may suggest that while TNFα may play a role in the acute phase of neuroinflammatory response at a mechanistic level, its serum levels might not be a good indicator for outcome.

This study integrated two circulating inflammatory molecules with suPAR and identified new potential prognostic biomarkers and therapeutic targets for the future development of diagnostic, prognostic, and treatment strategies for acute brain injuries. The lack of correlation between suPAR and clinical infection status in this cohort, as well as with CRP values, indicates that suPAR elevation in post-acute brain injury is more likely reflective of the neuroinflammatory response rather than secondary infectious complications.

## Limitations

Our study is not without limitations. The sample size, while adequate to demonstrate significant associations, limits the generalizability of our findings. The primary focus in this study was to develop a prognostic model; however, the limited number of healthy controls constrains the significance of suPAR’s diagnostic value. The limited control size reduces our capacity to account for natural variability in suPAR levels due to demographic factors such as age, sex, and underlying health conditions that may be present in larger populations. While prior studies indicate that suPAR levels were in the range of our results in healthy populations, a larger control group would provide a more accurate basis for comparison and help refine diagnostic utility. Thus, the results regarding suPAR’s diagnostic properties should be interpreted with these limitations in mind.

While multivariate analysis could further explore the independent predictive value of suPAR, the lack of significant correlations between suPAR and confounding variables such as age, sex, and infection status provide confidence in interpreting the LDA model, which was derived from Bonferroni-corrected biomarker candidates. Future studies with larger patient populations are needed to validate our results and explore the applicability of the suPAR and IL-1β prognostic model across different settings and populations.

Genetic, environmental, and medical factors were not analyzed in this study. These factors may also influence the prognosis after acute brain injuries. Additionally, our study’s observational nature precludes the establishment of causal relationships between biomarker levels and patient outcomes. Longitudinal studies that track biomarker levels over time could provide deeper insights into their dynamic changes and prognostic relevance.

## Conclusions

Our study contributes valuable evidence supporting the role of suPAR as a prognostic biomarker in acute brain injuries, with elevated levels associated with worse outcomes. The incorporation of IL-1β into a combined biomarker model further refines the prognostic accuracy offering a promising approach for improving patient management. This study paves the way for future research to explore the underlying common inflammatory mechanisms in recovery after different types of acute brain injuries.

## Supplementary Information

ESM 1 (PDF 13 kb)

ESM 2 (PDF 86 kb)

ESM 3 (PDF 60 kb)

ESM 4 (DOCX 30 kb)

ESM 5 (DOCX 259 kb)

ESM 6 (DOCX 33 kb)

## Data Availability

The anonymized data from this study can be made available upon request to qualified researchers who have obtained appropriate institutional review board (IRB) approval. Requests should be directed to the corresponding author (JK).

## References

[1] Wermer, Marieke J.H., Hieke Kool, Kees W. Albrecht, Gabriël J.E. Rinkel, and Aneurysm Screening after Treatment for Ruptured Aneurysms Study Group. 2007. Subarachnoid hemorrhage treated with clipping: Long-term effects on employment, relationships, personality, and mood. *Neurosurgery* 60: 91–97. 10.1227/01.NEU.0000249215.19591.86. 17228256 10.1227/01.NEU.0000249215.19591.86

[2] Ma, Vella, M.L. Crandall, and M.B. Patel. 2017. Acute Management of Traumatic Brain Injury. *The Surgical Clinics of North America* 97. 10.1016/j.suc.2017.06.003. 10.1016/j.suc.2017.06.003 PMC5747306 28958355

[3] Corps, Kara N., Theodore L. Roth, and Dorian B. McGavern. 2015. Inflammation and neuroprotection in traumatic brain injury. *JAMA Neurology* 72: 355–362. 10.1001/jamaneurol.2014.3558. 25599342 10.1001/jamaneurol.2014.3558 PMC5001842

[4] Hou, Duanlu, Chunjie Wang, Xiaofei Ye, Ping Zhong, and Wu. Danhong. 2021. Persistent inflammation worsens short-term outcomes in massive stroke patients. *BMC Neurology* 21: 62. 10.1186/s12883-021-02097-9. 33568099 10.1186/s12883-021-02097-9 PMC7874622

[5] Aisiku, Yamal, Pratik Doshi, Julia S. Benoit, Shankar Gopinath, Jerry C. Goodman, and Claudia S. Robertson. 2016. Plasma cytokines IL-6, IL-8, and IL-10 are associated with the development of acute respiratory distress syndrome in patients with severe traumatic brain injury. *Critical Care* 20: 288. 10.1186/s13054-016-1470-7. 27630085 10.1186/s13054-016-1470-7 PMC5024454

[6] Shichita, Takashi, Hiroaki Ooboshi, and Akihiko Yoshimura. 2023. Neuroimmune mechanisms and therapies mediating post-ischaemic brain injury and repair. *Nature Reviews Neuroscience* 24: 299–312. 10.1038/s41583-023-00690-0. 36973481 10.1038/s41583-023-00690-0

[7] Strogulski, Nathan R., Luis V. Portela, Brian M. Polster, and David J. Loane. 2023. Fundamental neurochemistry review: Microglial immunometabolism in traumatic brain injury. *Journal of Neurochemistry* 167: 129–153. 10.1111/jnc.15959. 37759406 10.1111/jnc.15959 PMC10655864

[8] Alsbrook, Diana L., Mario Di Napoli, Kunal Bhatia, José Biller, Sasan Andalib, Archana Hinduja, Roysten Rodrigues, et al. 2023. Neuroinflammation in acute ischemic and hemorrhagic stroke. *Current Neurology and Neuroscience Reports* 23: 407–431. 10.1007/s11910-023-01282-2. 37395873 10.1007/s11910-023-01282-2 PMC10544736

[9] Iadecola, Costantino, Marion S. Buckwalter, and Josef Anrather. 2020. Immune responses to stroke: Mechanisms, modulation, and therapeutic potential. *The Journal of Clinical Investigation* 130: 2777–2788. 10.1172/JCI135530. 32391806 10.1172/JCI135530 PMC7260029

[10] Simon, D.W., M.J. McGeachy, H. Bayır, R.S. Clark, D.J. Loane, and P.M. Kochanek. 2017. The far-reaching scope of neuroinflammation after traumatic brain injury. *Nature Reviews Neurology* 13. 10.1038/nrneurol.2017.13. 10.1038/nrneurol.2017.116 28776601

[11] Miller, Brandon A., Nefize Turan, Monica Chau, and Gustavo Pradilla. 2014. Inflammation, vasospasm, and brain injury after subarachnoid hemorrhage. *BioMed Research International* 2014: 384342. 10.1155/2014/384342. 25105123 10.1155/2014/384342 PMC4106062

[12] Thunø, Maria, Betina Macho, and Jesper Eugen-Olsen. 2009. suPAR: The molecular crystal ball. *Disease Markers* 27: 157–172. 10.3233/DMA-2009-0657. 19893210 10.3233/DMA-2009-0657 PMC3835059

[13] Donadello, Katia, Sabino Scolletta, Cecilia Covajes, and Jean-Louis Vincent. 2012. suPAR as a prognostic biomarker in sepsis. *BMC Medicine* 10: 2. 10.1186/1741-7015-10-2. 22221662 10.1186/1741-7015-10-2 PMC3275545

[14] Backes, Yara, Koenraad F. van der Sluijs, David P. Mackie, Frank Tacke, Alexander Koch, Jyrki J. Tenhunen, and Marcus J. Schultz. 2012. Usefulness of suPAR as a biological marker in patients with systemic inflammation or infection: A systematic review. *Intensive Care Medicine* 38: 1418–1428. 10.1007/s00134-012-2613-1. 22706919 10.1007/s00134-012-2613-1 PMC3423568

[15] Aronen, Anu, Janne Aittoniemi, Reetta Huttunen, Anssi Nikkola, Jussi Nikkola, Olli Limnell, Juhani Sand, and Johanna Laukkarinen. 2023. Plasma soluble urokinase-type plasminogen activator receptor (P-suPAR) in the diagnostics between malignant and non-malignant pancreatic lesions. *Pancreatology* 23: 213–217. 10.1016/j.pan.2022.12.012. 36596714 10.1016/j.pan.2022.12.012

[16] Sorio, Claudio, Andrea Mafficini, Federico Furlan, Stefano Barbi, Antonio Bonora, Giorgio Brocco, Francesco Blasi, Giorgio Talamini, Claudio Bassi, and Aldo Scarpa. 2011. Elevated urinary levels of urokinase-type plasminogen activator receptor (uPAR) in pancreatic ductal adenocarcinoma identify a clinically high-risk group. *BMC Cancer* 11: 448. 10.1186/1471-2407-11-448. 21999221 10.1186/1471-2407-11-448 PMC3213238

[17] Zhang, Qi, Le Li, Hongze Chen, Guangquan Zhang, Siqiang Zhu, Rui Kong, Hua Chen, Gang Wang, and Bei Sun. 2020. Soluble urokinase plasminogen activator receptor associates with higher risk, advanced disease severity as well as inflammation, and might serve as a prognostic biomarker of severe acute pancreatitis. *Journal of Clinical Laboratory Analysis* 34: e23097. 10.1002/jcla.23097. 31774228 10.1002/jcla.23097 PMC7083411

[18] Wu, Chung-Ze, Li-Chien Chang, Yuh-Feng Lin, Yi-Jen Hung, Dee Pei, Nain-Feng Chu, and Jin-Shuen Chen. 2015. Urokinase plasminogen activator receptor and its soluble form in common biopsy-proven kidney diseases and in staging of diabetic nephropathy. *Clinical Biochemistry* 48: 1324–1329. 10.1016/j.clinbiochem.2015.07.001. 26162494 10.1016/j.clinbiochem.2015.07.001

[19] Nusshag, Christian, Changli Wei, Eunsil Hahm, Salim S. Hayek, Jing Li, Beata Samelko, Christoph Rupp, et al. 2023. suPAR links a dysregulated immune response to tissue inflammation and sepsis-induced acute kidney injury. *JCI Insight* 8: e165740. 10.1172/jci.insight.165740. 37036003 10.1172/jci.insight.165740 PMC10132159

[20] Rasmussen, Line Jee, Jens Emil Hartmann, Vang Petersen, and Jesper Eugen-Olsen. 2021. Soluble Urokinase plasminogen activator receptor (suPAR) as a biomarker of systemic chronic inflammation. *Frontiers in Immunology* 12: 780641. 10.3389/fimmu.2021.780641. 34925360 10.3389/fimmu.2021.780641 PMC8674945

[21] Zhan, Kegang, Luhan Wang, Hao Lin, Xiaoyu Fang, Hong Jia, and Xiangyu Ma. 2023. Novel inflammatory biomarkers in the prognosis of COVID-19. *Therapeutic Advances in Respiratory Disease* 17: 17534666231199679. 10.1177/17534666231199679. 37727063 10.1177/17534666231199679 PMC10515606

[22] Onatsu, Juha, Mikko Taina, Pirjo Mustonen, Marja Hedman, Antti Muuronen, Otso Arponen, Miika Korhonen, Pekka Jäkälä, Ritva Vanninen, and Kari Pulkki. 2017. Soluble Urokinase-type plasminogen activator receptor predicts all-cause 5-year mortality in ischemic stroke and TIA. *In Vivo (Athens, Greece)* 31: 381–386. 10.21873/invivo.11070. 28438866 10.21873/invivo.11070 PMC5461448

[23] Hodges, Gethin, Stig Lyngbæk, Christian Selmer, Ole Ahlehoff, Simone Theilade, Thomas Berend Sehestedt, Ulrik Abildgaard, et al. 2020. SuPAR is associated with death and adverse cardiovascular outcomes in patients with suspected coronary artery disease. *Scandinavian Cardiovascular Journal* 54: 339–345. 10.1080/14017431.2020.1762917. 32400206 10.1080/14017431.2020.1762917

[24] Tp, Schmidt, W. Albanna, M. Weiss, M. Veldeman, C. Conzen, O. Nikoubashman, C. Blume, et al. 2022. The role of soluble Urokinase plasminogen activator receptor (suPAR) in the context of aneurysmal subarachnoid hemorrhage (aSAH)-a prospective observational study. *Frontiers in Neurology* 13. 10.3389/fneur.2022.841024. 10.3389/fneur.2022.841024 PMC8960720 35359651

[25] Frugier, Tony, Maria Cristina Morganti-Kossmann, David O’Reilly, and Catriona A. McLean. 2010. In situ detection of inflammatory mediators in post mortem human brain tissue after traumatic injury. *Journal of Neurotrauma* 27: 497–507. 10.1089/neu.2009.1120. 20030565 10.1089/neu.2009.1120

[26] Csuka, E., M.C. Morganti-Kossmann, P.M. Lenzlinger, H. Joller, O. Trentz, and T. Kossmann. 1999. IL-10 levels in cerebrospinal fluid and serum of patients with severe traumatic brain injury: Relationship to IL-6, TNF-alpha, TGF-beta1 and blood-brain barrier function. *Journal of Neuroimmunology* 101. 10.1016/s0165-5728(99)00148-4. 10.1016/s0165-5728(99)00148-4 10580806

[27] de Rivero Vaccari, J.P., G. Lotocki, O.F. Alonso, H.M. Bramlett, W.D. Dietrich, and R.W. Keane. 2009. Therapeutic neutralization of the NLRP1 inflammasome reduces the innate immune response and improves histopathology after traumatic brain injury. *Journal of Cerebral Blood Flow and Metabolism* 29. 10.1038/jcbfm.2009.46. 10.1038/jcbfm.2009.46 PMC2846547 19401709

[28] Viviani, Barbara, Mariaserena Boraso, Natalia Marchetti, and Marina Marinovich. 2014. Perspectives on neuroinflammation and excitotoxicity: A neurotoxic conspiracy? *Neurotoxicology* 43: 10–20. 10.1016/j.neuro.2014.03.004. 24662010 10.1016/j.neuro.2014.03.004

[29] Kim, Seung-Woo, Hahnbie Lee, Hye-Kyung Lee, Il-Doo Kim, and Ja-Kyeong Lee. 2019. Neutrophil extracellular trap induced by HMGB1 exacerbates damages in the ischemic brain. *Acta Neuropathologica Communications* 7: 94. 10.1186/s40478-019-0747-x. 31177989 10.1186/s40478-019-0747-x PMC6556959

[30] Smith, Craig J., Sharon Hulme, Andy Vail, Calvin Heal, Adrian R. Parry-Jones, Sylvia Scarth, Karen Hopkins, et al. 2018. SCIL-STROKE (subcutaneous Interleukin-1 receptor antagonist in ischemic stroke): A randomized controlled phase 2 trial. *Stroke* 49: 1210–1216. 10.1161/STROKEAHA.118.020750. 29567761 10.1161/STROKEAHA.118.020750

[31] Mastronardi, Claudio, Fiona Whelan, Ozlem A. Yildiz, Jonas Hannestad, David Elashoff, Samuel M. McCann, Julio Licinio, and Ma-Li Wong. 2007. Caspase 1 deficiency reduces inflammation-induced brain transcription. *Proceedings of the National Academy of Sciences of the United States of America* 104: 7205–7210. 10.1073/pnas.0701366104. 17409187 10.1073/pnas.0701366104 PMC1847598

[32] Linnerbauer, Mathias, Michael A. Wheeler, and Francisco J. Quintana. 2020. Astrocyte crosstalk in CNS inflammation. *Neuron* 108: 608–622. 10.1016/j.neuron.2020.08.012. 32898475 10.1016/j.neuron.2020.08.012 PMC7704785

[33] Diaz-Cañestro, Candela, Martin F. Reiner, Nicole R. Bonetti, Luca Liberale, Mario Merlini, Patricia Wüst, Heidi Amstalden, et al. 2019. AP-1 (activated Protein-1) transcription factor JunD regulates ischemia/reperfusion brain damage via IL-1β (interleukin-1β). *Stroke* 50: 469–477. 10.1161/STROKEAHA.118.023739. 30626291 10.1161/STROKEAHA.118.023739

[34] Hansson, Magnus J., and Eskil Elmér. 2023. Cyclosporine as therapy for traumatic brain injury. *Neurotherapeutics: The Journal of the American Society for Experimental NeuroTherapeutics* 20: 1482–1495. 10.1007/s13311-023-01414-z. 37561274 10.1007/s13311-023-01414-z PMC10684836

[35] Wu, Guanming, Eric Dawson, Adrian Duong, Robin Haw, and Lincoln Stein. 2014. ReactomeFIViz: A Cytoscape app for pathway and network-based data analysis. *F1000Research* 3: 146. 10.12688/f1000research.4431.2. 25309732 10.12688/f1000research.4431.1 PMC4184317

[36] Connolly, E. Sander, Alejandro A. Rabinstein, J. Ricardo Carhuapoma, Colin P. Derdeyn, Jacques Dion, Randall T. Higashida, Brian L. Hoh, et al. 2012. Guidelines for the management of aneurysmal subarachnoid hemorrhage: A guideline for healthcare professionals from the American Heart Association/american Stroke Association. *Stroke* 43: 1711–1737. 10.1161/STR.0b013e3182587839. 22556195 10.1161/STR.0b013e3182587839

[37] Carney, Nancy, Annette M. Totten, Cindy O’Reilly, Jamie S. Ullman, Gregory W.J. Hawryluk, Michael J. Bell, Susan L. Bratton, et al. 2017. Guidelines for the Management of Severe Traumatic Brain Injury, fourth edition. *Neurosurgery* 80: 6–15. 10.1227/NEU.0000000000001432. 27654000 10.1227/NEU.0000000000001432

[38] Powers, William J., Alejandro A. Rabinstein, Teri Ackerson, Opeolu M. Adeoye, Nicholas C. Bambakidis, Kyra Becker, José Biller, et al. 2019. Guidelines for the early Management of Patients with Acute Ischemic Stroke: 2019 update to the 2018 guidelines for the early Management of Acute Ischemic Stroke: A guideline for healthcare professionals from the American Heart Association/American Stroke Association. *Stroke* 50: e344–e418. 10.1161/STR.0000000000000211. 31662037 10.1161/STR.0000000000000211

[39] Girard, R., H.A. Zeineddine, J. Koskimäki, M.D. Fam, Y. Cao, C. Shi, T. Moore, et al. 2018. Plasma biomarkers of inflammation and angiogenesis predict cerebral cavernous malformation symptomatic hemorrhage or lesional growth. *Circulation Research* 122: 1716–1721. 10.1161/CIRCRESAHA.118.312680. 29720384 10.1161/CIRCRESAHA.118.312680 PMC5993629

[40] Lyne, S.B., R. Girard, J. Koskimaki, H.A. Zeineddine, D. Zhang, Y. Cao, Y. Li, et al. 2019. Biomarkers of cavernous angioma with symptomatic hemorrhage. *JCI Insight* 4: 31217347. 10.1172/jci.insight.128577. 10.1172/jci.insight.128577 PMC6629090 31217347

[41] Youden, W.J. 1950. Index for rating diagnostic tests. *Cancer* 3: 32–35. 10.1002/1097-0142(1950)3:1<32::aid-cncr2820030106>3.0.co;2-3. 15405679 10.1002/1097-0142(1950)3:1<32::aid-cncr2820030106>3.0.co;2-3

[42] Calster, Van, Ewout W. Ben, Laure Wynants Steyerberg, and Maarten van Smeden. 2023. There is no such thing as a validated prediction model. *BMC Medicine* 21: 70. 10.1186/s12916-023-02779-w. 36829188 10.1186/s12916-023-02779-w PMC9951847

[43] C, Bonferroni. 1936. Teoria statistica delle classi e calcolo delle probabilita. *Pubblicazioni del R Istituto Superiore di Scienze Economiche e Commericiali di Firenze* 8: 3–62.

[44] Motulsky, Harvey J., and Ronald E. Brown. 2006. Detecting outliers when fitting data with nonlinear regression - a new method based on robust nonlinear regression and the false discovery rate. *BMC Bioinformatics* 7: 123. 10.1186/1471-2105-7-123. 16526949 10.1186/1471-2105-7-123 PMC1472692

[45] Winnicki, Wolfgang, Gere Sunder-Plassmann, Gürkan Sengölge, Ammon Handisurya, Harald Herkner, Christoph Kornauth, Bernhard Bielesz, et al. 2019. Diagnostic and prognostic value of soluble Urokinase-type plasminogen activator receptor (suPAR) in focal segmental Glomerulosclerosis and impact of detection method. *Scientific Reports* 9: 13783. 10.1038/s41598-019-50405-8. 31551522 10.1038/s41598-019-50405-8 PMC6760112

[46] Chew-Harris, Janice, A. Sarah Appleby, Mark Richards, Richard W. Troughton, and Christopher J. Pemberton. 2019. Analytical, biochemical and clearance considerations of soluble urokinase plasminogen activator receptor (suPAR) in healthy individuals. *Clinical Biochemistry* 69: 36–44. 10.1016/j.clinbiochem.2019.05.010. 31129182 10.1016/j.clinbiochem.2019.05.010

[47] Wlazel, Rafal N., Katarzyna Szwabe, Agnieszka Guligowska, and Tomasz Kostka. 2020. Soluble urokinase plasminogen activator receptor level in individuals of advanced age. *Scientific Reports* 10: 15462. 10.1038/s41598-020-72377-w. 32963338 10.1038/s41598-020-72377-w PMC7508810

[48] Yu, Li, Wu Xiaoling, Hui Wang, Ding Long, Junhui Yang, and Yuanchao Zhang. 2014. Diagnostic and prognostic significance of suPAR in traumatic brain injury. *Neurology India* 62: 498–502. 10.4103/0028-3886.144439. 25387618 10.4103/0028-3886.144439

[49] Śmiłowski, K., M. Śmiłowski, R. Partyka, D. Kokocińska, and P. Jałowiecki. 2022. Personalised approach to diagnosing and managing ischemic stroke with a plasma-soluble Urokinase-type plasminogen activator receptor. *Journal of Personalized Medicine* 12. 10.3390/jpm12030457. 10.3390/jpm12030457 PMC8953259 35330458

[50] Kiiski, Heikki, Ville Jalkanen, Marika Ala-Peijari, Mari Hämäläinen, Eeva Moilanen, Jukka Peltola, and Jyrki Tenhunen. 2017. Plasma soluble Urokinase-type plasminogen activator receptor is not associated with neurological outcome in patients with aneurysmal subarachnoid hemorrhage. *Frontiers in Neurology* 8: 144. 10.3389/fneur.2017.00144. 28458650 10.3389/fneur.2017.00144 PMC5394110

[51] Hayek, Sever, Yi-An Ko, Howard Trachtman, Mosaab Awad, Shikha Wadhwani, Mehmet Aitintas, Changli Wei, Anna Hotton, et al. 2015. Soluble Urokinase receptor and chronic kidney disease. *The New England Journal of Medicine* 20: 373. 10.1056/NEJMoa1506362. 10.1056/NEJMoa1506362 PMC4701036 26539835

[52] Gustafsson, Anna, Lennart Ljunggren, Mikael Bodelsson, and Ingrid Berkestedt. 2012. The prognostic value of suPAR compared to other inflammatory markers in patients with severe Sepsis. *Biomarker Insights* 7: 39–44. 10.4137/BMI.S9460. 22550400 10.4137/BMI.S9460 PMC3329189

[53] Rodriguez, Juan, Cuicui Xie, Tao Li, Yanyan Sun, Yafeng Wang, Xu Yiran, Kenan Li, et al. 2020. Inhibiting the interaction between apoptosis-inducing factor and cyclophilin a prevents brain injury in neonatal mice after hypoxia-ischemia. *Neuropharmacology* 171: 108088. 10.1016/j.neuropharm.2020.108088. 32277944 10.1016/j.neuropharm.2020.108088

[54] Pan, Pengyu, Hengli Zhao, Xuan Zhang, Qiang Li, Qu Jie, Shilun Zuo, Fan Yang, et al. 2020. Cyclophilin a signaling induces pericyte-associated blood-brain barrier disruption after subarachnoid hemorrhage. *Journal of Neuroinflammation* 17: 16. 10.1186/s12974-020-1699-6. 31926558 10.1186/s12974-020-1699-6 PMC6954572

[55] Clausen, Fredrik, Anders Hånell, Charlotte Israelsson, Johanna Hedin, Ted Ebendal, Anis K. Mir, Hermann Gram, and Niklas Marklund. 2011. Neutralization of interleukin-1β reduces cerebral edema and tissue loss and improves late cognitive outcome following traumatic brain injury in mice. *The European Journal of Neuroscience* 34: 110–123. 10.1111/j.1460-9568.2011.07723.x. 21623956 10.1111/j.1460-9568.2011.07723.x

[56] Shao, Xuefei, Xiping Yang, Jun Shen, Sansong Chen, Xiaochun Jiang, Qifu Wang, and Qiang Di. 2020. TNF-α-induced p53 activation induces apoptosis in neurological injury. *Journal of Cellular and Molecular Medicine* 24: 6796–6803. 10.1111/jcmm.15333. 32344470 10.1111/jcmm.15333 PMC7299703

[57] Wu, Wei, Yi Guan, Gang Zhao, Fu Xi-Jia, Tie-Zhu Guo, Yue-Ting Liu, Xin-Liang Ren, Wei Wang, Han-Rui Liu, and Yun-Qian Li. 2016. Elevated IL-6 and TNF-α levels in cerebrospinal fluid of subarachnoid hemorrhage patients. *Molecular Neurobiology* 53: 3277–3285. 10.1007/s12035-015-9268-1. 26063595 10.1007/s12035-015-9268-1

