# Supplementary figures and images for: Soluble Urokinase-Type Plasminogen Activator Receptor and Inflammatory Biomarker Response with Prognostic Significance after Acute Neuronal Injury – a Prospective Cohort Study
Source: Inflammation. 2024 Nov 14;48(4):2217–29. doi: 10.1007/s10753-024-02185-1 (PMC12336084; doi:10.1007/s10753-024-02185-1)

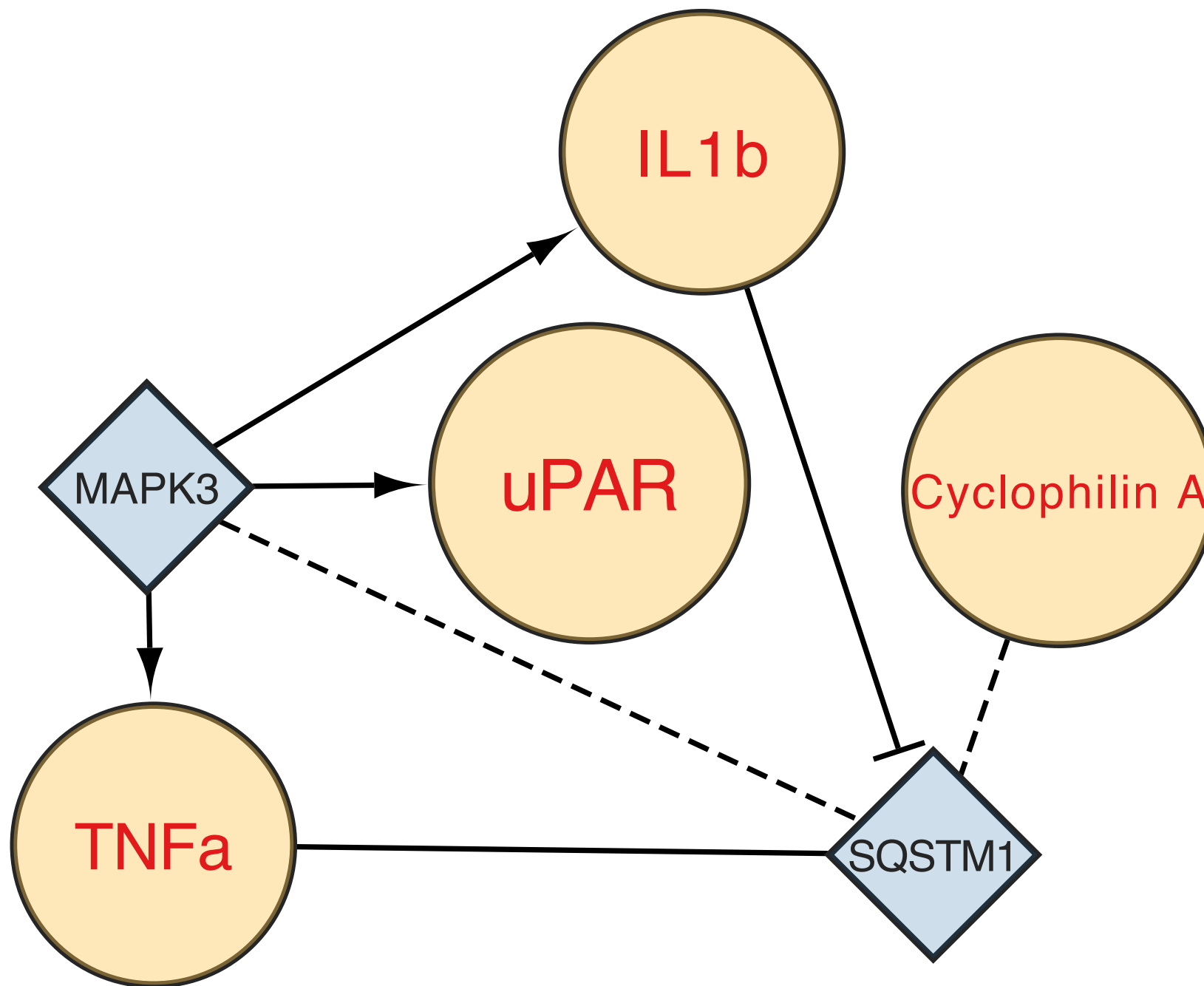

Supplement: Supplementary file 1 — (PDF 13 kb) [file 10753_2024_2185_MOESM1_ESM.pdf]

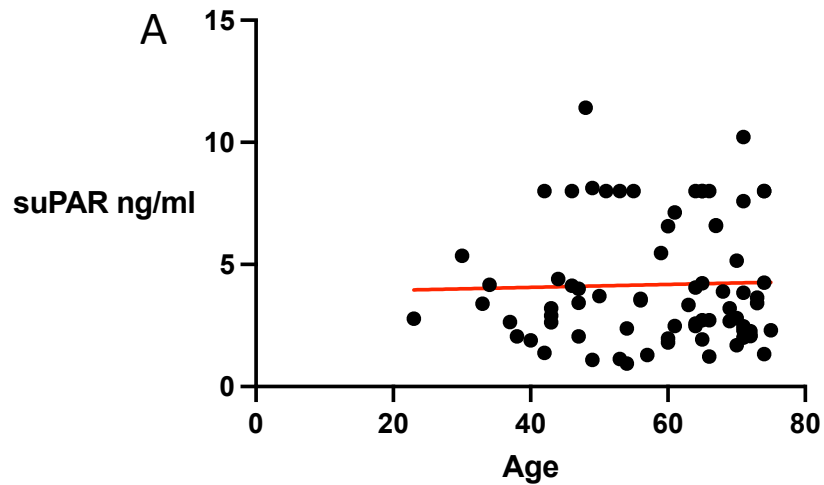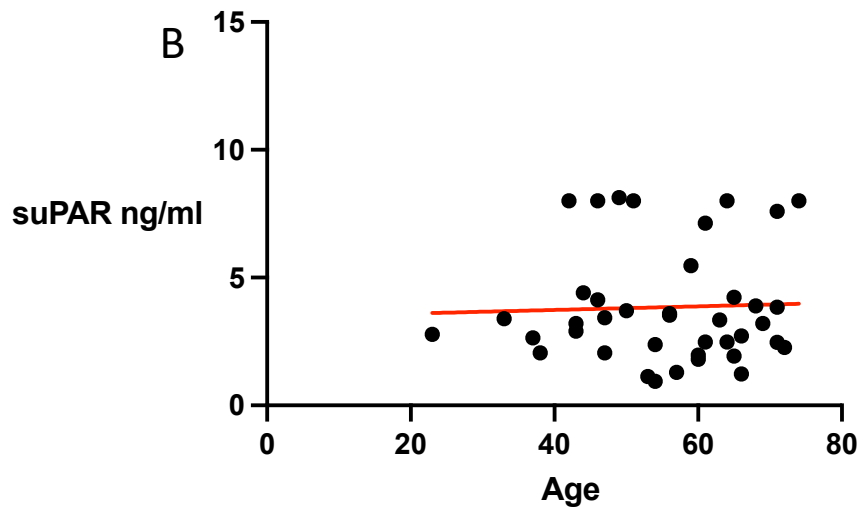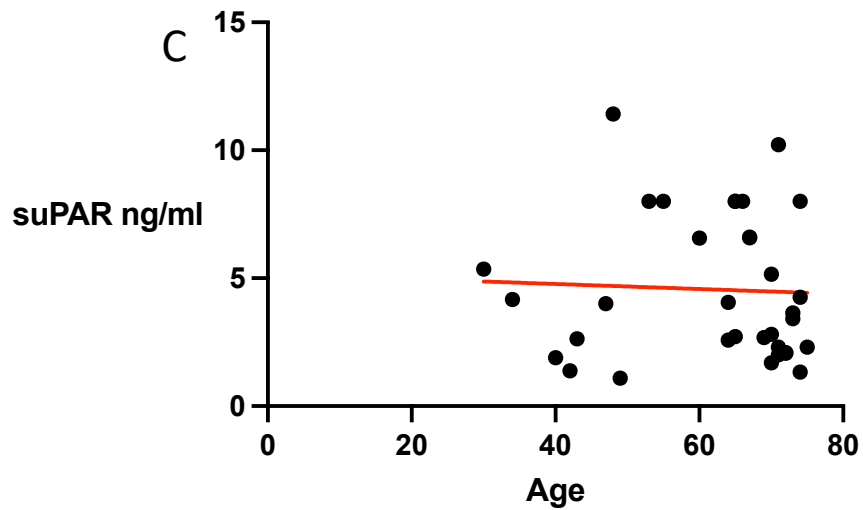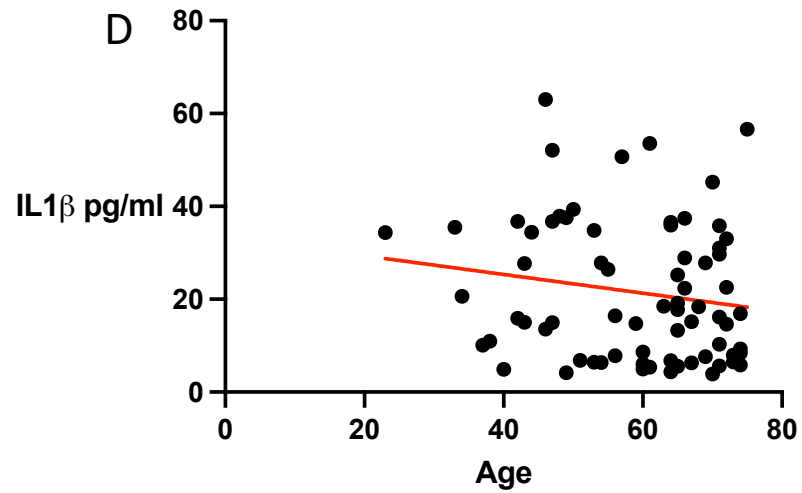

Supplement: Supplementary file 2 — (PDF 86 kb) [file 10753_2024_2185_MOESM2_ESM.pdf]

**A) Favorable outcome group differences**

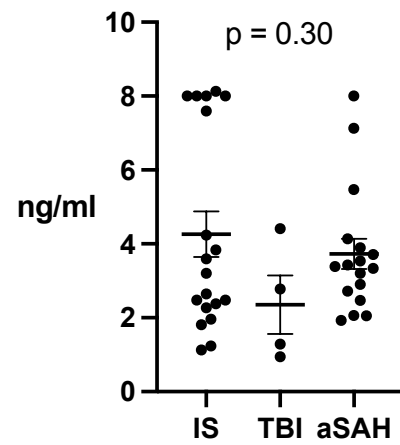

**B) Unfavorable outcome group differences**

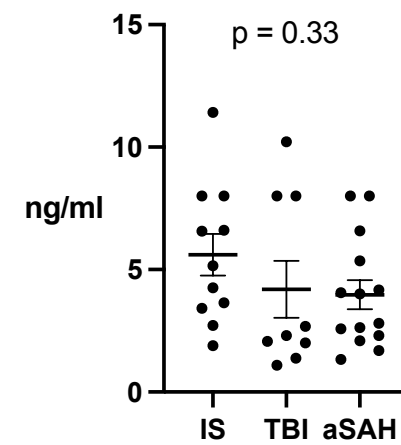

Supplement: Supplementary file 3 — (PDF 60 kb) [file 10753_2024_2185_MOESM3_ESM.pdf]
