# Supplementary material for: Soluble Urokinase-Type Plasminogen Activator Receptor and Inflammatory Biomarker Response with Prognostic Significance after Acute Neuronal Injury – a Prospective Cohort Study
Source: Inflammation. 2024 Nov 14;48(4):2217–29. doi: 10.1007/s10753-024-02185-1 (PMC12336084; doi:10.1007/s10753-024-02185-1)
Supplement: Supplementary file 4 — (DOCX 30 kb) [file 10753_2024_2185_MOESM4_ESM.docx]

**Table S1. Characteristics of the study population (n=74)**

| **VARIABLE** | **STATISTICS** |
| --- | --- |
| **Age in years** |  |
| Mean±SD | 58.4±12.7 |
| Min–Max | 23.0–75.0 |
| Median (IQR) | 62.0 (48.0–70.0) |
| **Sex** |  |
| Male (%) | 39 (52.7) |
| Female (%) | 35 (47.3) |
| **General health** |  |
| No diseases (%) | 15 (20.3) |
| Mild diseases but normal health (%) | 32 (43.2) |
| Significant diseases that affect general health (%) | 25 (33.8) |
| Unable to work due to poor general health (%) | 2 (2.7) |
| **Earlier brain injuries** |  |
| No (%) | 65 (87.8) |
| Yes (%) | 9 (12.2) |
| **Alcohol abuse** |  |
| Missing | 1 (1.4) |
| No (%) | 53 (72.6) |
| Yes (%) | 20 (27.4) |
| **Drug abuse** |  |
| Missing | 1 (1.4) |
| No (%) | 71 (97.3) |
| Yes (%) | 2 (2.7) |
|  |  |
|  |  |
|  |  |
|  |  |
|  |  |

| Variable | Statistics |
| --- | --- |
| **Smoking** |  |
| Missing (%) | 4 (5.4) |
| No (%) | 39 (55.7) |
| Yes (%) | 31 (44.3) |
| **Type of brain injury** |  |
| Aneurysmal subarachnoid hemorrhage (%) | 31 (41.9) |
| Traumatic brain injury (%) | 13 (17.6) |
| Ischemic stroke (%) | 30 (40.5) |
| **Location of brain injury** |  |
| Missing (%) | 10 (13.5) |
| Frontal (%) | 15 (23.4) |
| Parietal (%) | 1 (1.6) |
| Temporal (%) | 35 (54.7) |
| Occipital (%) | 1 (1.6) |
| Brainstem (%) | 3 (4.7) |
| Multifocal (%) | 9 (14.1) |
| **Location of infarction** |  |
| MCA (%) | 20 (27.0) |
| ACA (%) | 5 (6.8) |
| PCA (%) | 5 (6.8) |
| Basilar (%) | 2 (2.7) |
| Lacunar (%) | 4 (5.4) |
| Vertebral (%) | 2 (2.7) |
| No infarction (%) | 36 (48.6) |
|  |  |
|  |  |
|  |  |
|  |  |
|  |  |

| Variables | Statistics |
| --- | --- |
| **Volume of infarction (ml)** |  |
| Mean±SD | 47.6±70.3 |
| Min–Max | 0.1–302.5 |
| Median (IQR) | 24.6 (2.8–52.7) |
| **Midline shift (mm)** |  |
| Mean±SD | 2.6±4.5 |
| Min–Max | 0.0–20.0 |
| Median (IQR) | 0.0 (0.0–5.0) |
| **Volume of aSDH (ml)** |  |
| Mean±SD | 56.3±45.9 |
| Min–Max | 0.4–122.3 |
| Median (IQR) | 46.0 (10.9–101.8) |
| **Basal cisterns compression** |  |
| No (%) | 39 (52.7) |
| Absent (%) | 8 (10.8) |
| Compressed (%) | 27 (36.5) |
| **IVH component** |  |
| No (%) | 46 (62.2) |
| Yes (%) | 28 (37.8) |
| **ICH component** |  |
| No (%) | 42 (56.8) |
| Yes (%) | 32 (43.2) |
|  |  |
|  |  |
|  |  |
|  |  |
|  |  |

| Variables | Statistics |
| --- | --- |
| **Hemorrhagic infarction** |  |
| No (%) | 67 (90.5) |
| Yes (%) | 7 (9.5) |
| **Neuroworsening** |  |
| No (%) | 38 (51.4) |
| Yes (%) | 36 (48.6) |
| **Type of stroke** |  |
| Cardiogenic (%) | 6 (20.7) |
| Thrombosis (%) | 10 (34.5) |
| Cryptic (%) | 13 (44.8) |
| **Toast** |  |
| Atherosclerosis of main arteries (%) | 5 (19.2) |
| Cardiogenic embolism (%) | 6 (23.1) |
| Disease of small vessels (%) | 1 (3.8) |
| Other known etiology (%) | 2 (7.7) |
| Unknown (%) | 12 (46.2) |
| **Modified Fisher scale** |  |
| 1 (%) | 1 (3.2) |
| 2 (%) | 3 (9.7) |
| 3 (%) | 4 (12.9) |
| 4 (%) | 23 (74.2) |
|  |  |
|  |  |
|  |  |
|  |  |
|  |  |

| Variables | Statistics |
| --- | --- |
| **Hunt & Hess** |  |
| 1 (%) | 10 (32.3) |
| 2 (%) | 6 (19.4) |
| 3 (%) | 4 (12.9) |
| 4 (%) | 5 (16.1) |
| 5 (%) | 6 (19.4) |
| **WFNS** |  |
| 1 (%) | 10 (32.3) |
| 2 (%) | 8 (25.8) |
| 3 (%) | 2 (6.5) |
| 4 (%) | 2 (6.5) |
| 5 (%) | 9 (29.0) |
| **Clinical DCI** |  |
| No (%) | 21 (67.7) |
| Yes (%) | 10 (32.3) |
| **Alcohol involved** |  |
| Missing | 2 (2.7) |
| No (%) | 66 (91.7) |
| Yes (%) | 6 (8.3) |
| **NIHSS** |  |
| Mean±SD | 7.4±6.3 |
| Min–Max | 0.0–20.0 |
| Median (IQR) | 6.0 (1.5–13.5) |
|  |  |

| Variables | Statistics |
| --- | --- |
| **Location of Aneurysm** |  |
| Anterior circulation (%) | 28 (90.3) |
| Posterior circulation (%) | 3 (9.7) |
| **Location of Aneurysm specific** |  |
| Missing (%) | 43 (58.1) |
| ACA (%) | 2 (6.5) |
| AcomA (%) | 10 (32.3) |
| MCA (%) | 6 (19.4) |
| Basilar (%) | 2 (6.5) |
| Pcom (%) | 3 (9.7) |
| PICA (%) | 1 (3.2) |
| ICA (%) | 7 (22.6) |
| **Aneurysm Deformed** |  |
| No (%) | 13 (41.9) |
| Yes (%) | 18 (58.1) |
| **Width of neck (mm)** |  |
| Mean±SD | 3.0±1.5 |
| Min–Max | 1.3–8.6 |
| Median (IQR) | 2.9 (2.0–3.5) |
|  |  |
|  |  |
|  |  |
|  |  |

| Variables | Statistics |
| --- | --- |
| **Type of Aneurysm** |  |
| Saccular (%) | 19 (61.3) |
| Fusiform (%) | 12 (38.7) |
| **GCS at scene** |  |
| Mean±SD | 11.8±4.4 |
| Min–Max | 3.0–15.0 |
| Median (IQR) | 15.0 (8.0–15.0) |
| **ICP monitoring** |  |
| No (%) | 45 (60.3) |
| Yes (%) | 29 (39.7) |
| **Hemicraniectomy** |  |
| No (%) | 69 (94.6) |
| Yes (%) | 5 (6.8) |
| **ICP therapy drainage** |  |
| No (%) | 47 (63.5) |
| Yes (%) | 27 (36.5) |
|  |  |
|  |  |
|  |  |
|  |  |
|  |  |

| Variables | Statistics |
| --- | --- |
| **Diagnosis of acute hydrocephalus** |  |
| No (%) | 59 (79.7) |
| Yes (%) | 15 (20.3) |
| **Diagnosis of shunt dependent hydrocephalus** |  |
| No (%) | 62 (83.6) |
| Yes (%) | 12 (16.4) |
| **Neuropsychological symptoms** |  |
| Missing (%) | 13 (17.6) |
| No (%) | 27 (44.3) |
| Yes (%) | 34 (55.7) |
| **mRS** |  |
| 0 (%) | 13 (17.6) |
| 1 (%) | 10 (13.5) |
| 2 (%) | 17 (23.0) |
| 3 (%) | 6 (8.1) |
| 4 (%) | 9 (12.2) |
| 5 (%) | 3 (4.1) |
| 6 (%) | 16 (21.6) |
|  |  |
|  |  |
|  |  |
|  |  |
|  |  |
|  |  |

| Variables | Statistics |
| --- | --- |
| **aSDH thickness (mm)** |  |
| Mean±SD | 12.1±6.1 |
| Min–Max | 4.5–20.0 |
| Median (IQR) | 12.0 (6.0–18.0) |
| **aSDH location** |  |
| Left (%) | 7 (53.8) |
| Right (%) | 6 (46.2) |
