# Supplementary material for: Soluble Urokinase-Type Plasminogen Activator Receptor and Inflammatory Biomarker Response with Prognostic Significance after Acute Neuronal Injury – a Prospective Cohort Study
Source: Inflammation. 2024 Nov 14;48(4):2217–29. doi: 10.1007/s10753-024-02185-1 (PMC12336084; doi:10.1007/s10753-024-02185-1)
Supplement: Supplementary file 5 — (DOCX 259 kb) [file 10753_2024_2185_MOESM5_ESM.docx]

**Soluble urokinase-type plasminogen activator receptor and inflammatory biomarker response with prognostic significance after acute neuronal injury – a prospective cohort study**

Antti Sajanti^1^, Santtu Hellström^1^, Carolyn Bennett^2^, Abhinav Srinath^2^, Aditya Jhaveri^2^, Ying Cao^3^, Riikka Takala^4^, Janek Frantzén^1^, Fredrika Koskimäki^5^, Johannes Falter^6^, Seán B. Lyne^7^, Tomi Rantamäki^8^, Jussi P. Posti^1^, Susanna Roine^5^, Miro Jänkälä^9^, Jukka Puolitaival^9^, Sulo Kolehmainen^10^, Romuald Girard^2^, Melissa Rahi^1^, Jaakko Rinne^1^, Eero Castrén^10^, Janne Koskimäki^1,9,10^

^1^Neurocenter, Department of Neurosurgery, Turku University Hospital and University of Turku, P.O. Box 52, Hämeentie 11, FI-20521, Turku, Finland.

^2^Neurovascular Surgery Program, Section of Neurosurgery, The University of Chicago Medicine and Biological Sciences, 5841 S. Maryland, Chicago, IL 60637, US.

^3^Department of Radiation Oncology, Kansas University Medical Center, Kansas City, KS 66160, USA

^4^Perioperative Services, Intensive Care and Pain Medicine, Turku University Hospital and University of Turku, POB 52, 20521, Turku, Finland.

^5^Neurocenter, Acute Stroke Unit, Turku University Hospital, P.O. Box 52, FI-20521, Turku, Finland

^6^Department of Neurosurgery, University Medical Center of Regensburg, Regensburg, Germany

^7^Department of Neurosurgery, Brigham and Women’s Hospital, Harvard Medical School, Boston, Massachusetts.

^8^Laboratory of Neurotherapeutics, Molecular and Integrative Biosciences Research Programme, Faculty of Biological and Environmental Sciences and Drug Research Program, Division of Pharmacology and Pharmacotherapy, Faculty of Pharmacy, University of Helsinki, Finland

^9^Department of Neurosurgery, Oulu University Hospital, Box 25, 90029 OYS, Finland.

^10^Neuroscience Center, HiLIFE, University of Helsinki, Box 63, 00014 Helsinki, Finland.

**Corresponding author:**

Janne Koskimäki, MD, PhD, Associate Professor

Neurocenter, Department of Neurosurgery, Turku University Hospital and University of Turku, P.O. Box 52 (Hämeentie 11), FI-20521, Turku, Finland.

E-mail: jankosk@utu.fi

**Supplemental results**

**Network analysis**

The supplemental *figure S1* illustrates the interaction network of selected inflammatory molecules IL-1β, suPAR, TNFα, and Cyclophilin A, highlighting their interconnected roles in biological functional network. IL-1β and TNFα interact with the MAPK3, indicating its critical role in inflammatory responses. suPAR is connecting to both IL-1β and TNFα. Additionally, suPAR is influenced by MAPK3. Cyclophilin A is linked to SQSTM1, integrating it into the broader inflammatory network. The network analysis, depicting direct (solid lines) and indirect (dashed lines) interactions, underscores the interconnectedness of these biomarkers, supporting their combined study to enhance understanding of inflammatory responses in acute brain injuries.

**Biomarkers**

In the supplemental analysis of our study, Pearson correlation analysis was conducted to explore the relationship between patients' age, suPAR, and IL-1β concentrations (*Figure S2*). No significant correlation was found between suPAR concentration and age (r = 0.03010, R² = 0.0009, p = 0.7990), indicating that suPAR levels are independent of age. In contrast, IL-1β showed a trend towards a negative correlation with age (r = -0.01657, R²=0.02745, p = 0.1673), suggesting a potential decrease in IL-1β levels with increasing age, though this trend was not statistically significant.

We also sought to understand the behavior of suPAR as a neuroinflammatory biomarker response in different acute brain injury conditions (*Figure S3*). Conducting an ANOVA, we found no significant differences in suPAR levels between patients suffering from ischemic stroke (IS), aneurysmal subarachnoid hemorrhage (aSAH), and traumatic brain injury (TBI), irrespective of the outcomes being favorable (p = 0.30) or unfavorable (p = 0.33). This homogeneity in suPAR expression suggests a common response that might be involved across these clinical scenarios, supporting the consolidation of these groups into a larger single cohort for further analysis. Indeed, we observed a trend indicating increasing suPAR concentrations from favorable to unfavorable outcomes within each individual disease group (IS, TBI, aSAH). However, likely due to the limited sample sizes in these subgroups, these trends did not achieve statistical significance (IS p = 0.21, TBI p = 0.34, aSAH p = 0.24). This pattern suggests a common relationship between suPAR levels and the severity of outcomes, as showed in pooled analyses.

Pooling the data from these groups served to enhance our sample size, bolstering the statistical robustness of our study and allowing for a more generalized interpretation of suPAR as a potential biomarker. The consistency in suPAR levels across diverse brain injuries further implies a homogeneity of the inflammatory response brain injury, indicative of suPAR's role as a universal marker in the neuroinflammatory process following acute brain injuries. This uniform response, coupled with the increased power of a larger sample, reinforces the potential utility of suPAR in clinical prognostication and may have implications for future therapeutic strategies.

**Supplemental figures**

**Figure S1. Focused functional interaction network of urokinase-type plasminogen activator receptor (uPAR) and associated proteins analyzed with ReactomeFIViz in Cytoscape.** Red label indicates inputted proteins. Black label indicates linker proteins. Arrow = activation, flat bar = inhibition, line = direction undetermined, dashed line = association. MAPK3 = mitogen-activated protein kinase 3, SQSTM1 = sequestosome-1.

**Figure S2. Pearson correlation analysis between patients’ age, suPAR and IL-1β. A)** No correlation was identified between suPAR concentration and age (r=0.03010, R^2^=0.0009, p=0.7990). **B)** No correlation was identified between suPAR concentration and age in the favorable outcome group (r=0.03790, R^2^=0.0014, p=0.8164). **C)** No correlation was identified between suPAR concentration and age in the unfavorable outcome group (r=-0.0448, R^2^=0.0020, p=0.8013).  **D)** A trend towards negative correlation was identified between IL1-β concentration and age (r=-0.01657, R^2^=0.02745, p=0.1673).

**Figure S3. Soluble urokinase-type plasminogen activator receptor (suPAR) concentrations in different brain injury groups.** A) Comparison of suPAR levels in the favorable outcome group across ischemic stroke (IS), aneurysmal subarachnoid hemorrhage (aSAH), and traumatic brain injury (TBI), showing no significant difference (p = 0.30). B) Analysis of suPAR concentrations in the unfavorable outcome group among IS, aSAH, and TBI patients, also indicating no statistically significant variance (p = 0.33). ANOVA, data are represented as mean ± SEM.
